# Multi-omics decodes host-specific and environmental microbiome interactions in sepsis

**DOI:** 10.3389/fmicb.2025.1618177

**Published:** 2025-06-26

**Authors:** Jiamin Lu, Wen Zhang, Yuzhou He, Mei Jiang, Zhankui Liu, Jirong Zhang, Lanzhi Zheng, Bingzhi Zhou, Jielian Luo, Chenming He, Yunan Shan, Runze Zhang, KaiLiang Fan, Bangjiang Fang, Chuanqi Wan

**Affiliations:** ^1^Longhua Hospital, Shanghai University of Traditional Chinese Medicine, Shanghai, China; ^2^Institute of Acute and Critical Care, Shanghai University of Traditional Chinese Medicine, Shanghai, China; ^3^The Second Affiliated Hospital of Zhejiang Chinese Medical University, Hangzhou, China; ^4^Tianjin University of Traditional Chinese Medicine, Tianjin, China; ^5^The First Affiliated Hospital of Zhejiang Chinese Medical University, Hangzhou, China; ^6^School of Traditional Chinese Medicine, Hubei University of Chinese Medicine, Wuhan, China; ^7^The Affiliated Hospital of Shandong University of Traditional Chinese Medicine, Jinan, China

**Keywords:** bioinformatics tools, comparative genome analysis, microbiome, multi-omics, sepsis

## Abstract

Sepsis is a life-threatening organ dysfunction caused by a dysregulated host response to infection, and its pathogenesis involves complex interactions between the host and the microbiome. The integration of multi-omics has important value in revealing the mechanism of host-microbiome interaction. It is a key tool for promoting accurate diagnosis and guiding dynamic treatment strategies in sepsis. However, multi-omics data integration faces technical challenges, such as data heterogeneity and platform variability, as well as analytical hurdles, such as the “curse of dimensionality.” Fortunately, researchers have developed two integration strategies: data-driven and knowledge-guided approaches, which employ various dimensionality reduction techniques and integration methods to handle multi-omics datasets. This review discusses the applications of multi-omics technologies in host-microbiome interactions in sepsis, highlighting their potential in identifying novel diagnostic biomarkers and developing personalized and dynamic treatment strategies. It also summarizes commonly used systems biology resources and computational tools for data integration; the review outlines the challenges in this field and proposes potential directions for future studies.

## Graphical abstract

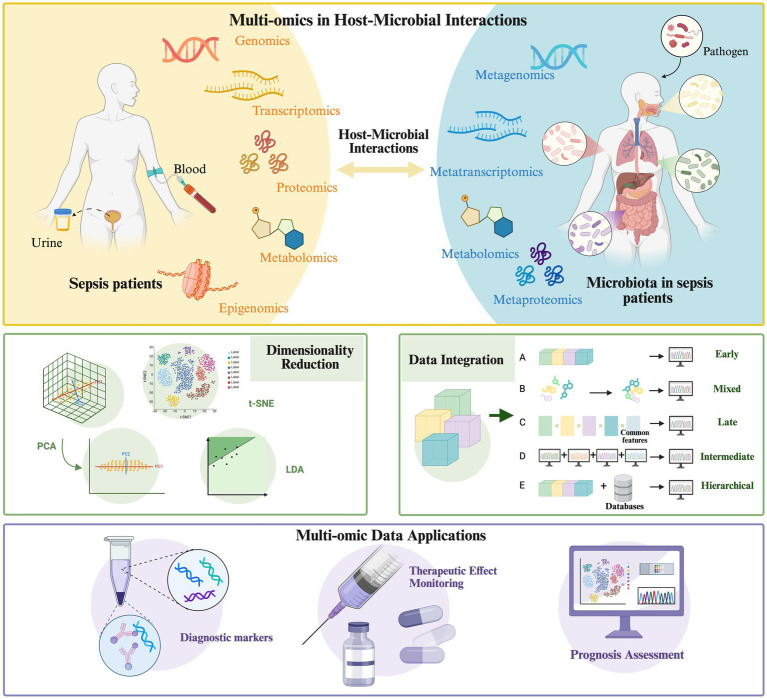

## 1 Introduction

Sepsis is a life-threatening disease with a global impact, posing a severe threat to humans and representing one of the significant challenges in global healthcare. It is characterized by high incidence and mortality rates, with data indicating that it causes approximately 5.3 million deaths annually worldwide (Evans et al., 2021; Fleischmann et al., 2016). Although the ability to treat patients with sepsis is improved, the mortality rate of sepsis remains unacceptably high. Therefore, identifying novel pathogenic mechanisms is critical for improving outcomes in sepsis patients. Sepsis can be classified by severity into sepsis and septic shock, with the latter representing the severe stage of sepsis. This study encompasses research related to both sepsis and septic shock (Singer et al., 2016).

Over the past two decades, research on the microbiome has grown exponentially. The human microbiome consists of diverse microorganisms, including bacteria, viruses, fungi, and archaea, which coexist symbiotically with the human host and play a crucial role in maintaining homeostasis (Koppel and Balskus, 2016). Dysbiosis of the microbial community is both a consequence and a contributing factor in the pathogenesis of sepsis, occupying a critical position in its progression. This imbalance can affect the host’s immune response, metabolic processes, and barrier function, thereby influencing the outcome of sepsis (Weersma et al., 2020). Therefore, understanding the complex interactions between the host and microbiome in sepsis is of significant value for identifying novel diagnostic biomarkers and developing personalized and dynamic therapeutic strategies.

Genomic sepsis studies can elucidate associations between host genetic variations and susceptibility (Rayzan et al., 2023). Metagenomics reveals changes in microbial diversity and function (Kalantar et al., 2022). Transcriptomics aids in identifying sepsis biomarkers and infection types (Chen et al., 2024). Proteomics uncovers host immune responses and metabolic remodeling processes (Miao et al., 2021). Metabolomics assists in sepsis diagnosis and therapeutic monitoring (Pandey, 2024). Epigenomics clarifies epigenetic mechanisms by which microbes influence host immunity (Córneo et al., 2021). However, single-omics analyses provide an incomplete perspective. Therefore, integrating multi-omics approaches enables a comprehensive and systematic dissection of biological systems. First, this integration helps identify novel biomarkers of host–microbe interactions in sepsis, such as specific microbes and metabolites, thereby enhancing diagnostic accuracy (Khan et al., 2019). Second, it facilitates the development of personalized therapeutic strategies, such as modulating gut microbiota and precision drug administration (Isac et al., 2024). Thirdly, multi-omics technologies enable real-time therapeutic efficacy monitoring, predict patient outcomes, and assist clinicians in promptly adjusting treatment regimens (Sun et al., 2023).

The reproducibility of multi-omics data, combined with advanced modeling techniques, facilitates comprehensive and integrative analysis of these complex systems (Santiago-Rodriguez and Hollister, 2021). Public datasets enable the reuse of high-quality data, while machine learning and network analysis tools uncover patterns and relationships within them. Integrative multi-omics approaches, such as multi-omics factor analysis and systems biology modeling, allow for simultaneous analysis across multiple biological levels, revealing correlations and causal relationships between omics layers. We posit that in sepsis research, there is a need for further synthesis and integration of multi-omics approaches and technologies, as well as a holistic perspective on host-microbe interrelationships and interactions.

## 2 Omics studies of host and microbiome in sepsis

Due to its high incidence and mortality, sepsis remains a priority health concern highlighted by the World Health Organization (WHO) (Rudd et al., 2020). As such, omics technologies have increasingly been applied to explore sepsis’s pathogenesis and therapeutic targets. Kurian et al. (2024) demonstrated that research on sepsis utilizing omics technologies has shown a growing trend in publications from the European Union and the United Kingdom, with microbiology being one of the primary research directions. Notably, studies in this field have proliferated since 2000, with 1,608 articles published between 2011 and May 2023. These findings provide new insights into the pathophysiology of sepsis and contribute to rapid diagnosis, targeted therapy, and personalized medicine (Ahmed, 2022).

### 2.1 Genomics and macrogenomics

Genomics elucidates the relationship between host genetic variations and sepsis susceptibility, therapeutic responses, and clinical outcomes by analyzing the genetic material in host somatic cells. Standard detection techniques include whole genome sequencing (WGS), whole exome sequencing (WES), and single nucleotide polymorphism (SNP) genotyping (Ahmed et al., 2021). Several studies have used WES to reveal the impact of rare immune-deficiency gene variants on sepsis susceptibility in pediatric patients (Rayzan et al., 2023; Borghesi et al., 2020). However, the static nature of genomics limits in-depth exploration of the dynamic course of sepsis. Future research needs to shift toward studying phenotypes and dynamic gene expression.

Metagenomics enables sequencing microbial genomes (archaea, bacteria, viruses, and fungi) present in samples, providing critical insights into microbial diversity and functional potential (Almeida et al., 2019). Common approaches include amplicon-based marker gene analysis (e.g., 16S rRNA gene sequencing) and metagenomic shotgun sequencing. It will be interesting to mention that besides 16S rRNA gene, which is useful for identifying bacterial species on a broader taxonomic scale, more exact identification requires further genetic techniques, such as Nanopore sequencing, which produces long sequencing reads (Abedini-Nassab, 2017; Ying et al., 2013). This 3rd generation sequencing technology is able to sequence the whole 16S rRNA gene and not just some of its variable regions. Metagenomic sequencing has revealed significant alterations in the fecal microbiota of sepsis patients, such as increased abundances of *Bacillota*, *Bacteroidota*, *Lactobacillaceae*, and opportunistic pathogens such as *Klebsiella* spp. and *Escherichia-Shigella* in septic rats (Sun et al., 2020). Functional analyses of phyla such as *Bacteroidota* and *Proteobacteria* help elucidate the dysbiosis associated with sepsis (Daliri et al., 2021). Additionally, metagenomic data on the nasal microbiota, including genera such as *Staphylococcus* spp., *Moraxella*, and *Streptococcus*, enhance predictive diagnostic capabilities for patients with lower respiratory tract infections (Li et al., 2022). The 16S rRNA gene sequencing technique provides critical microbiomic evidence for the subtyping and diagnosis of sepsis, facilitating the elucidation of the microbial mechanisms underlying sepsis susceptibility. Liu et al. (2021) employed this technology to demonstrate a strong correlation between gut microbiota composition and sepsis subtype susceptibility, proposing two dysbiosis models: ICU Enterotype I is characterized by a predominance of *Bacteroides* and unclassified *Enterobacteriaceae*, with hosts more prone to progressing to septic shock. ICU Enterotype II is dominated by *Enterococcus* spp., corresponding to hosts predominantly exhibiting a sepsis phenotype without concomitant shock. The application of whole-genome sequencing (WGS) for precision monitoring microbial genomes facilitates accurate clinical diagnosis of infectious microorganisms, enhancing diagnostic accuracy in clinical settings (Huang et al., 2019).

### 2.2 Transcriptomics and metatranscriptomics

The mRNA offers insights into the pathophysiology of sepsis patients. Whole-blood transcriptomics identifies biologically homogeneous subgroups by revealing differentially expressed genes and detects dynamic changes in sepsis (Saxena et al., 2024). In recent years, transcriptomics-based biomarkers have shown great potential in diagnosing, disease monitoring, and prognosis evaluation of sepsis. Some studies have conducted transcriptomic analyses on whole-blood RNA samples from sepsis patients and combined these with bioinformatics methods to identify nine genes, such as LRG1, ELANE, and TP53, as potential biomarkers for sepsis (Gong et al., 2020). The newly developed IMX-BVN-1 classifier, utilizing 29 preselected host mRNAs, employs a neural network to distinguish between bacterial and viral infections. This approach offers a novel method for rapid diagnosis in sepsis patients (Mayhew et al., 2020). Transcriptomics not only aids in rapidly differentiating infection types but also guides personalized sepsis treatment. Cano-Gamez et al. (2022) constructed a cross-platform transcriptomic reference map using transcriptomic data from different technical platforms. They proposed an immune dysfunction score (SRSq) for sepsis patient stratification, reflecting the degree of immune dysregulation and predicting clinical outcomes, thus providing new directions for early sepsis diagnosis and personalized treatment. Two subclasses of pediatric infectious shock patients were identified through genome-wide expression profiling based on whole blood RNA sequencing, providing information for clinical decision-making in sepsis (Yang et al., 2023). Kalantar et al. (2022) combined host transcriptional profiling with broad-range metagenomic pathogen detection from nucleic acids to develop a novel diagnostic tool for sepsis.

Metatranscriptomics can compensate for the limitations of metagenomics by analyzing microbial transcription levels to reveal their metabolic and functional states (Santiago-Rodriguez et al., 2015). Metagenomics focuses on the genetic potential of microbial communities, while metatranscriptomics provides insights into the actual gene expression and functional activities of microbes under specific conditions (Franzosa et al., 2014). This approach is beneficial for understanding microbial responses to environmental changes and their dynamic metabolic processes. Wang et al. (2023) performed metatranscriptomic sequencing on bronchoalveolar lavage fluid (BALF) from COVID-19 patients, analyzing host transcriptomic profiles, viral, bacterial, and fungal content, as well as virulence factors. They found that SARS-CoV-2, human β-herpesvirus, and phyla such as Proteobacteria and *Bacillota* were highly represented. They revealed a significant correlation between microbial composition and host immune responses.

### 2.3 Proteomics

Proteomic profiling of host body fluids such as blood and urine, using high-resolution mass spectrometry technologies such as LC–MS/MS to analyze post-translationally modified proteins, can reveal the immune responses and metabolic remodeling processes in sepsis (Duong and Lee, 2023). Proteomic profiling, based on shotgun proteomics and utilizing tandem mass spectrometry (MS) to identify species-specific peptides, distinguishes microbes at the amino acid level. This technique, when applied to analyze clinical samples from sepsis patients, aids in diagnosing sepsis infections (van Houten et al., 2018). However, its clinical application is limited due to technical complexity and high costs. Wang C. et al. (2021) performed a quantitative proteomic analysis of neutrophil proteins in the blood of sepsis patients and analyzed the blood microbiota. They found significant changes in different stages of sepsis, with interactions between microbiota changes and immune cell functional alterations. Bacterial genera were identified as potential predictive biomarkers for sepsis, providing new research and clinical management directions. Liu et al. (2019) performed proteomic analysis of fecal samples from sepsis patients and combined it with 16S rDNA sequencing and metabolomics. They found that sepsis leads to significant changes in gut microbiota protein expression, closely related to immune responses and coagulation function. These findings highlight the potential of gut microbiota proteins as biomarkers for sepsis.

### 2.4 Metabolomics

Metabolomics aids in sepsis diagnosis by detecting host metabolites (e.g., in blood, urine, and tissues) and microbial metabolites (e.g., SCFAs and amino acids), revealing host–microbe interactions and their impact on health and disease. Commonly used techniques include nuclear magnetic resonance (NMR) and liquid chromatography-mass spectrometry (LC–MS) (Bauermeister et al., 2022). Analysis of gut fecal metabolites can serve as an early non-invasive diagnostic tool for sepsis, particularly for late-onset sepsis in preterm infants caused by Gram-negative bacteria. Specific metabolic markers, such as ethyl acetate and cyclopentane, are associated with Gram-negative late-onset sepsis. Additionally, octanal has been identified as a unique metabolic marker for late-onset sepsis caused by coagulase-negative staphylococci (Frerichs et al., 2023).

Metabolomics data can be utilized to monitor therapeutic effects, particularly by analyzing specific metabolites correlated with disease outcomes, thereby supporting personalized treatment strategies. For instance, metabolomic analysis in mice demonstrated that gut microbiota-derived short-chain fatty acids (SCFAs), including acetate, propionate, and butyrate, were significantly associated with reduced levels of *Lactobacillus* and *Bifidobacterium* in the microbiota of mice with *Klebsiella pneumonia* induced pneumonic sepsis. These findings highlight the therapeutic potential of SCFAs in sepsis (Wu et al., 2020b). Wang et al. (2024b) found that changes in the gut microbiota and metabolites of sepsis patients are closely related to changes in serum vitamin levels. Vitamin B9 protects the intestinal barrier and reduces the severity of sepsis by altering the type and abundance of gut microbiota and upregulating the concentration of microbiota metabolites, thereby increasing the expression of intestinal barrier-related genes. Stewart et al. (2017) conducted metabolomic profiling of fecal samples from preterm infants with late-onset sepsis, combined with 16S rRNA sequencing. Their findings suggest that increased prebiotic oligosaccharides and the growth of *Bifidobacterium* in the gut exert a protective effect. While metabolomics can provide information on the correlation between metabolites and diseases, a single technique cannot trace the origin of metabolites (host, microbial, or co-microbial). Additionally, real-time metabolite detection technologies in critically ill clinical patients remain underdeveloped.

### 2.5 Epigenomics

In sepsis-related host-microbiota interactions, the inflammatory state promotes the influence of diverse microbes, particularly gut microbiota, on host cellular transcriptional programs and immune cell function. This process occurs through epigenetic mechanisms such as the production of epigenetic substrates and enzymatic regulators, modulation of DNA methylation and histone modifications, and regulation of non-coding RNA. These processes ultimately disrupt immune function and induce organ dysfunction (Woo and Alenghat, 2022; Binnie et al., 2020). In the gut microbiota, *Bacteroides fragilis*, *Clostridium perfringens*, and *Lactobacillus acidophilus*, along with their metabolites, enhance DNA methylation levels in T-lymphocytes, induce the production of various cytokines, mitigate the inflammatory response during sepsis, regulate the balance of immune cells, and maintain intestinal immune homeostasis (Heffernan et al., 2021). SCFAs produced by the gut microbiota induce histone post-translational modifications (PTMs), thereby influencing gene expression and other cellular processes (Gates et al., 2024). These modifications regulate protein activity and affect cellular signaling events in sepsis, helping to control cytokine storms and immunosuppression during the disease process (Zhang L. et al., 2023).

Some pathogens induce epigenetic changes in the host, promoting inflammation, regulating immune cell function, and modulating responses to microbial infections (Ma et al., 2024). This process provides new insights for preventing sepsis caused by pathogenic microorganisms. In sepsis caused by *Staphylococcus aureus* in mice, DNMT3A, an essential enzyme for DNA methylation, is suppressed in blood leukocytes *in vivo* and in macrophages and neutrophils *in vitro*. This suppression affects IL-10 regulation, impacting host immune responses. Through a DNA methylation-dependent mechanism, this process influences the host’s resistance to MRSA (Mba Medie et al., 2019).

## 3 Interaction between host immune system and microbiome in sepsis

### 3.1 Shaping of microbial communities by host immunity

**Figure 1:**
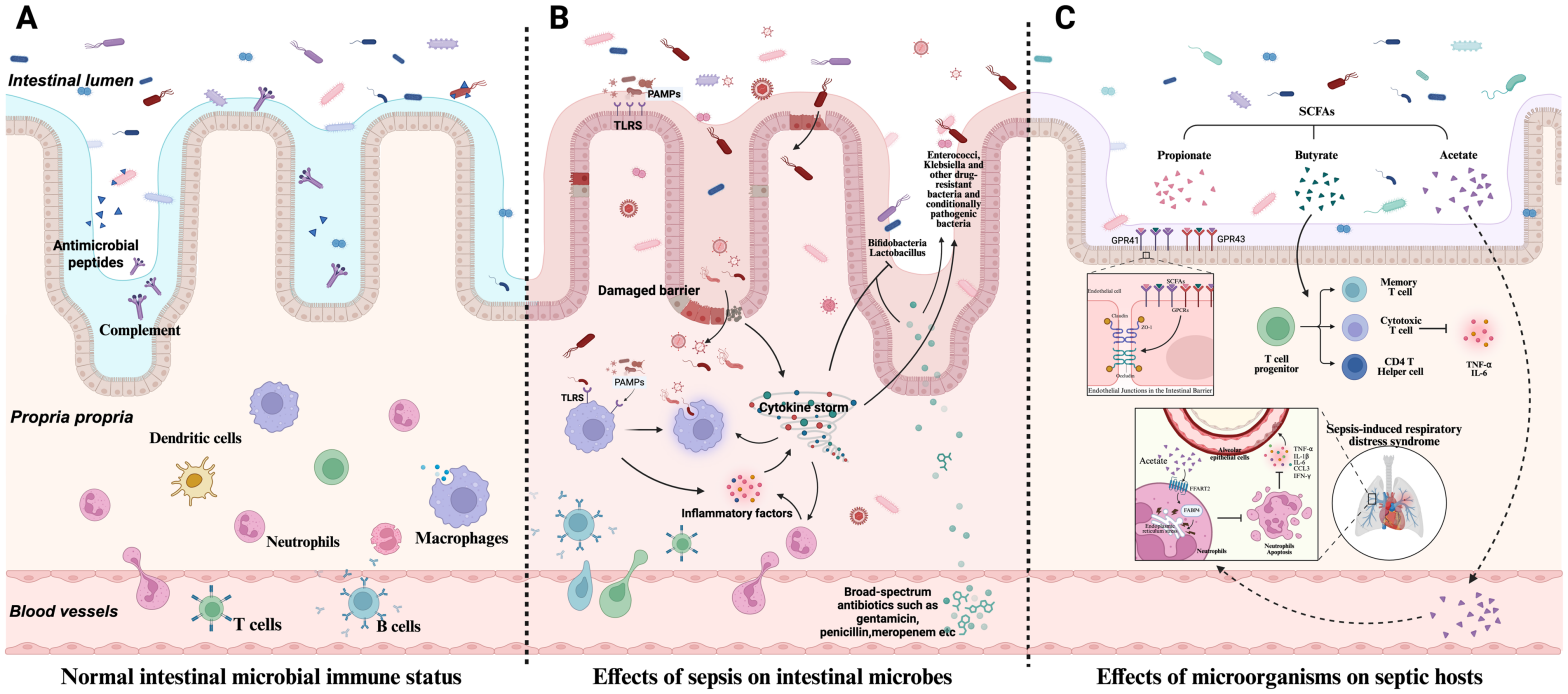
Host microbiome interactions in sepsis. **(A)** Under normal conditions, the host relies on innate immunity mechanisms (such as antimicrobial peptide secretion, complement activation, etc.) and adaptive immunity (such as antibody production, immune cell-mediated killing, etc.) to maintain the balance of intestinal microbial community. **(B)** The strong immune response triggered by sepsis breaks the original ecological balance of intestinal microbial community. **(C)** Microorganisms influence sepsis development and host immune response.

The host immune system is critical in regulating microbial communities, as shown in Figures 1A,B. Under normal conditions, the host maintains intestinal microbiota homeostasis through innate immune mechanisms (e.g., secretion of antimicrobial peptides, complement activation) and adaptive immune mechanisms (e.g., antibody production, immune cell-mediated cytotoxicity) (Potruch et al., 2022). Sepsis-induced robust immune responses disrupt the ecological balance of the gut microbiota. Toll-like receptors (TLRs) recognize pathogen-associated molecular patterns (PAMPs) from microorganisms, triggering intracellular signaling pathways that stimulate macrophages to secrete pro-inflammatory cytokines. This inflammatory response alters the gut microenvironment and affects microbial survival.

Additionally, therapeutic interventions such as antibiotic treatment lead to the massive elimination of susceptible bacteria, allowing drug-resistant or opportunistic pathogens to overgrow, thereby promoting bacterial translocation and the displacement of microbial metabolites (Thazha et al., 2019; Piccioni et al., 2024; Kalra et al., 2022). Broad-spectrum antibiotics reduce microbial diversity and increase the abundance of *Enterococcus* species, which is directly associated with an increased risk of sepsis (Kang and Thomas, 2021). In infants treated with broad-spectrum antibiotics (e.g., penicillin, gentamicin, or amoxiclav) for suspected early-onset neonatal sepsis, a significant reduction in *Bifidobacterium* abundance and an increase in *Klebsiella* spp. and *Enterococcus* abundance are observed, profoundly impacting the development of the gut microbiota (Reyman et al., 2022).

### 3.2 Modulation of host immunity by the microbiome

The microbiota plays a dual role in sepsis: it is both a pathogenic factor and a critical modulator of disease progression and host immune responses, as shown in Figure 1C. Microbiota diversity significantly impacts the immunophenotype and mortality of sepsis. In septic mice with high gut microbiota β-diversity, survival rates are improved considerably, accompanied by enhanced CD4+T cell responses. Conversely, reduced diversity impairs immune function, leading to uncontrolled inflammation and increased mortality (Fay et al., 2019). Furthermore, alterations in the gut microbiota before disease onset increase susceptibility to sepsis through multiple mechanisms, such as the expansion of pathogenic gut bacteria, activation of pro-inflammatory immune responses, and reduced production of beneficial microbial metabolites (Adelman et al., 2020).

Microbial dysbiosis can lead to host immune dysregulation during sepsis. The microbiota primarily modulates host immune responses through metabolites such as SCFAs and molecular patterns, thereby influencing the progression of sepsis (Mann et al., 2024). SCFAs (e.g., acetate, propionate, and butyrate) regulate epithelial barrier function, mucosal, and systemic immunity by signaling through G protein-coupled receptors (GPCRs) such as GPR41 and GPR43 or by modulating histone deacetylase activity, thereby alleviating sepsis-associated inflammation (Wang et al., 2024a). Butyrate enhances histone H3 acetylation at the Foxp3 promoter and other conserved non-coding sequences, inducing the differentiation of gut Treg cells and reducing TNF-*α* and IL-6 levels (Furusawa et al., 2013). Acetate lowers neutrophil apoptosis via the FFAR2 pathway. It downregulates FABP4 through the endoplasmic reticulum stress pathway, modulating neutrophil apoptosis, increasing inflammatory factors in lung epithelial cells, and aggravating sepsis-induced respiratory distress syndrome (Xuan et al., 2024).

### 3.3 Association of microbiome with sepsis-related organ dysfunction

#### 3.3.1 Gut microbiome and intestinal barrier function

Intestinal microbiota maintains gut barrier integrity through multiple mechanisms, including promoting the expression and stability of tight junction proteins, enhancing mucosal barrier function, and regulating intestinal immune cell homeostasis. Tight junction proteins (e.g., occludin, claudin family proteins) are central components of the mechanical barrier, forming tight intercellular junctions that effectively prevent the translocation of intestinal bacteria, toxins, and other harmful substances across the mucosal barrier into the bloodstream (Serek and Oleksy-Wawrzyniak, 2021).

In sepsis, microbial dysbiosis can compromise intestinal barrier function. On the one hand, the overgrowth of pathogenic bacteria directly erodes intestinal epithelial cells, disrupting tight junction structures. This process leads to increased tight junction proteins claudin-2 and JAM-A and decreased expression of claudin-5 and occludin, significantly increasing intestinal permeability (Yoseph et al., 2016). On the other hand, an imbalance in the gut microbiota impairs the gut’s immune regulatory functions, weakening the ability of immune cells to clear pathogens and further exacerbating intestinal barrier damage. Once the intestinal barrier is compromised, intestinal bacteria and their metabolites can translocate in large quantities into the mesenteric lymph nodes, portal venous system, and even directly into the bloodstream, causing systemic infection and serving as a critical trigger for sepsis (Kullberg et al., 2021). For example, D-lactic acid, produced by gut commensal bacteria, is transported via the portal vein to the liver, where it is essential for maintaining the integrity of the intra-vascular firewall mediated by Kupffer cells, enabling the capture and elimination of circulating pathogens (McDonald et al., 2020). Oral supplementation of SCFAs activates G-protein-coupled receptor 43, enhancing macrophage phagocytosis of *Klebsiella pneumoniae* (Wu et al., 2020a).

#### 3.3.2 Effects of microbial changes in sepsis on other organs

Sepsis disrupts the gut microbiota and causes dysbiosis in the lung microbiota. In patients with sepsis or acute respiratory distress syndrome (ARDS), alveolar microbial diversity decreases, and the lung microbiota network shifts from being dominated by beneficial commensal bacteria (e.g., *Streptococcus salivarius* and *Streptococcus oralis*) to being dominated by gastrointestinal and periodontal pathogens (Lu et al., 2024). This altered microbial network can induce immunosuppression by promoting the expression of the EGFR gene and suppressing the expression of BST2 and HLA-C genes. The “gut-lung axis” modulates pulmonary immune responses through microbial-associated molecular patterns (MAMPs) and metabolites. Oral/pharyngeal bacteria (e.g., *Klebsiella pneumoniae* and *Pseudomonas aeruginosa*) and gut bacteria (e.g., *Enterococcus* and *Klebsiella* spp.) can translocate to the lungs, causing infections and further disrupting systemic immunity (Lee and Banerjee, 2020).

Sepsis-associated neuroinflammation is closely linked to the imbalance of the gut-brain axis. Gut microbiota and SCFA metabolic disorders play a key role in sepsis-associated encephalopathy (SAE). SCFA supplementation can alleviate cognitive impairment and neuroinflammation in SAE mice (Li et al., 2023). Moreover, metformin has therapeutic potential for sepsis-related neuroinflammation by modulating the gut microbiota and metabolites, but its specific metabolic mechanisms require further validation (Zhao et al., 2022).

## 4 Strategies and methods of integrative omics in sepsis research

### 4.1 Necessity and challenges of multi-omics data integration

#### 4.1.1 Necessity of a comprehensive understanding of biological systems

Sepsis involves complex interactions between the host and pathogens, with interconnected information from multiple levels, including the microbiome, genome, transcriptome, proteome, and metabolome (Mangioni et al., 2020). For example, changes in the composition and function of the gut microbiota are closely linked to the host’s immune response during sepsis. A single-omics approach cannot fully elucidate this intricate relationship. In studies on *Staphylococcus epidermidis* in neonatal sepsis, the pathogenic potential and molecular mechanisms of this bacterium in the sepsis microbiome can only be fully understood by combining the open and diverse nature of its genome with the expression of virulence genes at the transcriptomic level. This integrated approach provides a basis for precision diagnosis and treatment (Joubert et al., 2022).

#### 4.1.2 Technical and analytical challenges of data integration

In clinical sepsis research, the three common goals of using omics are to investigate host responses, develop diagnostic methods, and identify clinically relevant clusters (Schuurman et al., 2021). However, the integration of multi-omics data faces numerous technical barriers. On the one hand, different types of omics data vary in data structure, measurement scale, and noise level (Chen et al., 2023; Wang W. et al., 2021). For example, 16S rRNA sequencing, used to analyze bacterial community structure, produces relatively sparse data. Researchers can consider integrating bacterial, viral, fungal, and protozoan communities to obtain more comprehensive information, but this increases complexity (Zhou et al., 2022). Metagenomic sequencing provides more comprehensive genetic information but generates large volumes of complex data, differing from nucleic acid-based omics data.

On the other hand, differences in data acquisition platforms and experimental conditions also complicate integration, as batch effects often interfere with data consistency and comparability. Analytically, the “curse of dimensionality” from high-dimensional data challenges traditional statistical methods, which struggle to handle the complexity of multi-omics data. Models are prone to overfitting, lack generalizability, and fail to apply to new datasets (Gliozzo et al., 2024).

### 4.2 Multi-omics integration strategy

In recent years, multi-omics research has developed numerous integration strategies to address challenges. These strategies can be broadly categorized into data-driven and knowledge-guided approaches, with a comparison provided in Table 1. Data-driven strategies focus on extracting meaningful patterns and relationships directly from raw omics data. This approach is highly flexible and suitable for complex datasets commonly encountered in multi-omics studies.

**Table 1:** Differences between data-methods and knowledge-guided methods.

Difference	Data-driven methods	Knowledge-guided learning methods
Basic idea	Directly extract patterns and relationships from raw data without relying on prior biological hypotheses.	Utilize existing biological knowledge and databases to simplify data complexity using prior information.
Common techniques/tools	PCA, t-SNE, NMDS, LDA, WGCNA, correlation analysis, network fusion (e.g., SNF)	GSEA, GSVA, pathway annotation based on KEGG/Reactome, specialized databases such as CAZy and VFDB
Advantages	Flexible and unbiased, capable of uncovering novel patterns; well-suited for handling large-scale, high-dimensional data.	Offers strong biological interpretability by integrating data into functional modules, reducing noise, and identifying known key pathways.
Limitations	Requires a high sample size; some patterns discovered may be challenging to interpret.	Database update frequency may affect accuracy depending on existing knowledge and potentially missing novel or unrecorded biological mechanisms.
Integration stage	Commonly used in early to intermediate integration stages (e.g., data concatenation, feature transformation, network construction).	Typically applied in later stages, using functional annotation and pathway aggregation to interpret and validate analysis results.

However, since extracted correlations may not always align with biological reality, more studies have begun incorporating external biological knowledge for guidance. By leveraging existing biological or functional knowledge (typically stored in multiple databases in network format, as shown in Table 2), knowledge-guided approaches effectively reduce multi-omics data’s complexity and enhance the integrated results’ biological significance and interpretability (Li W. et al., 2024). For example, in microbiome data integration, many microbial genes can be aggregated into functional modules or pathway levels using functional families from the PATRIC database (Davis et al., 2020) or gene families from the KEGG database (Kanehisa et al., 2023). Additionally, specialized databases focused on microbial genome annotation and comparison, such as IMG/M (Integrated Microbial Genomes & Microbiomes) (Chen et al., 2019) and MicroScope (Vallenet et al., 2020), can also be employed for this purpose. Additionally, for specific functional domains, specialized databases such as CARD (Alcock et al., 2020) for antibiotic resistance genes, VFDB (Zhou et al., 2025) for bacterial virulence factors, and mobileOG-db (Brown et al., 2022) for mobile genetic elements can be integrated. This approach provides a more comprehensive understanding of microbial community functional characteristics and interactions. Furthermore, bioinformatics tools such as PICRUSt (Langille et al., 2013), Piphillin (Narayan et al., 2020), PUMAA (Mitchell et al., 2020), and iVikodak (Nagpal et al., 2018) enable functional prediction based on 16S rRNA data, addressing functional research needs in cases where metagenomic sequencing is unavailable or resource-limited.

**Table 2:** Representative databases for various types of biological knowledge.

Database name	Full name	Biological knowledge	Source
UniProt-KB	UniProt Knowledgebase (UniProt Consortium, 2025)	Sequence and functional information on proteins	https://www.uniprot.org/
KEGG	Kyoto Encyclopedia of genes and genomes (Kanehisa and Goto, 2000)	Molecular interaction, reaction, and relation networks	https://www.genome.jp/kegg/pathway.html
Reactome	Reactome pathway database (Croft et al., 2011)	Signaling and metabolic pathways	https://reactome.org/
STRING	Search Tool for the retrieval of interacting genes/proteins (Szklarczyk et al., 2023)	Protein–protein interaction networks	https://string-db.org/
PathBank	PathBank (Wishart et al., 2024)	Metabolic, signaling, disease, drug, and physiological pathways	https://www.pathbank.org/
Pathway Commons	Pathway Commons (Cerami et al., 2011)	Biological pathway and interactions: biochemical reactions; gene regulatory networks; protein, nucleic acid, small molecule interactions	https://www.pathwaycommons.org/
BioCyc	BioCyc Pathway/Genome Database Collection (Karp et al., 2019)	Metabolic pathways, regulatory networks	https://biocyc.org/
WikiPathways	WikiPathways (Agrawal et al., 2024)	Signaling pathways	https://www.wikipathways.org/
GRNdb	Gene Regulatory Network database (Fang et al., 2021)	Gene regulatory networks among transcription factors and genes	http://www.grndb.com/
BioGRID	Biological General Repository for Interaction Datasets (Oughtred et al., 2021)	Protein and genetic interactions	https://thebiogrid.org/
IID	Integrated Interactions Database (Kotlyar et al., 2022)	Protein–protein interaction	http://ophid.utoronto.ca/iid
Harmonize	Harmonize (Rouillard et al., 2016)	Integrative gene and protein expression data across various tissues and conditions	http://amp.pharm.mssm.edu/Harmonizome
Cat RAPID	Computational Analysis of Targets of RNA-Protein Interactions and Discovery (Armaos et al., 2021)	RNA-protein interaction prediction	http://service.tartaglialab.com/page/catrapid_group
RBPDB	RNA-Binding Protein DataBase (Cook et al., 2011)	RNA-binding protein recognition motifs and their target interactions	http://rbpdb.ccbr.utoronto.ca/
RPISeq	RNA–Protein Interaction Sequence-based Predictor (Muppirala et al., 2011)	RNA-protein interactions	http://pridb.gdcb.iastate.edu/RPISeq/
RNAct	RNA - Critical Targets (Lang et al., 2019)	Protein-RNA interactions	http://rnact.crg.eu
StarBase	StarBase (Li et al., 2014)	miRNA-target, RNA–RNA interactions	http://starbase.sysu.edu.cn/
MetaCyc	Metabolic Pathways From all Domains of Life (Caspi et al., 2006)	Metabolic pathways	http://MetaCyc.org/
PHI-base	Pathogen Host Interactions Database (Winnenburg et al., 2006)	fungal and Oomycete pathogenicity genes-host interactions	http://www.phi-base.org/
ConsensusPathDB	ConsensusPathDB (The ConsensusPathDB Interaction Database, 2013)	Integrative database for molecular interactions	http://consensuspathdb.org/
PATRIC	Pathosystems Resource Integration Center (Wattam et al., 2014)	Microbial genomes and associated functional annotations	https://www.patricbrc.org/
IMG/M	The Integrated Microbial Genomes & Microbiomes (Chen et al., 2019)	Multi-Source Microbial Genomes and Metagenomes prediction and functional annotation	https://img.jgi.doe.gov/m/
MicroScope	MicroScope (Vallenet et al., 2020)	Functional annotation of microbial species genes and genomic regions, metabolic network reconstruction, and post-genomic experiments	https://www.genoscope.cns.fr/agc/microscope

Key signaling molecules in the host’s transcriptomic and proteomic data can be utilized to construct detailed gene regulatory networks by leveraging gene expression and regulatory information integrated in Harmonizome (Rouillard et al., 2016), as well as RNA-protein interaction data provided by catRAPID (Armaos et al., 2021), RBPDB (Cook et al., 2011), and RNAct (Lang et al., 2019). In practice, data-driven and knowledge-derived DR can complement each other, providing a more comprehensive understanding of the critical interactions between the host and microbes in sepsis.

**Figure 2:**
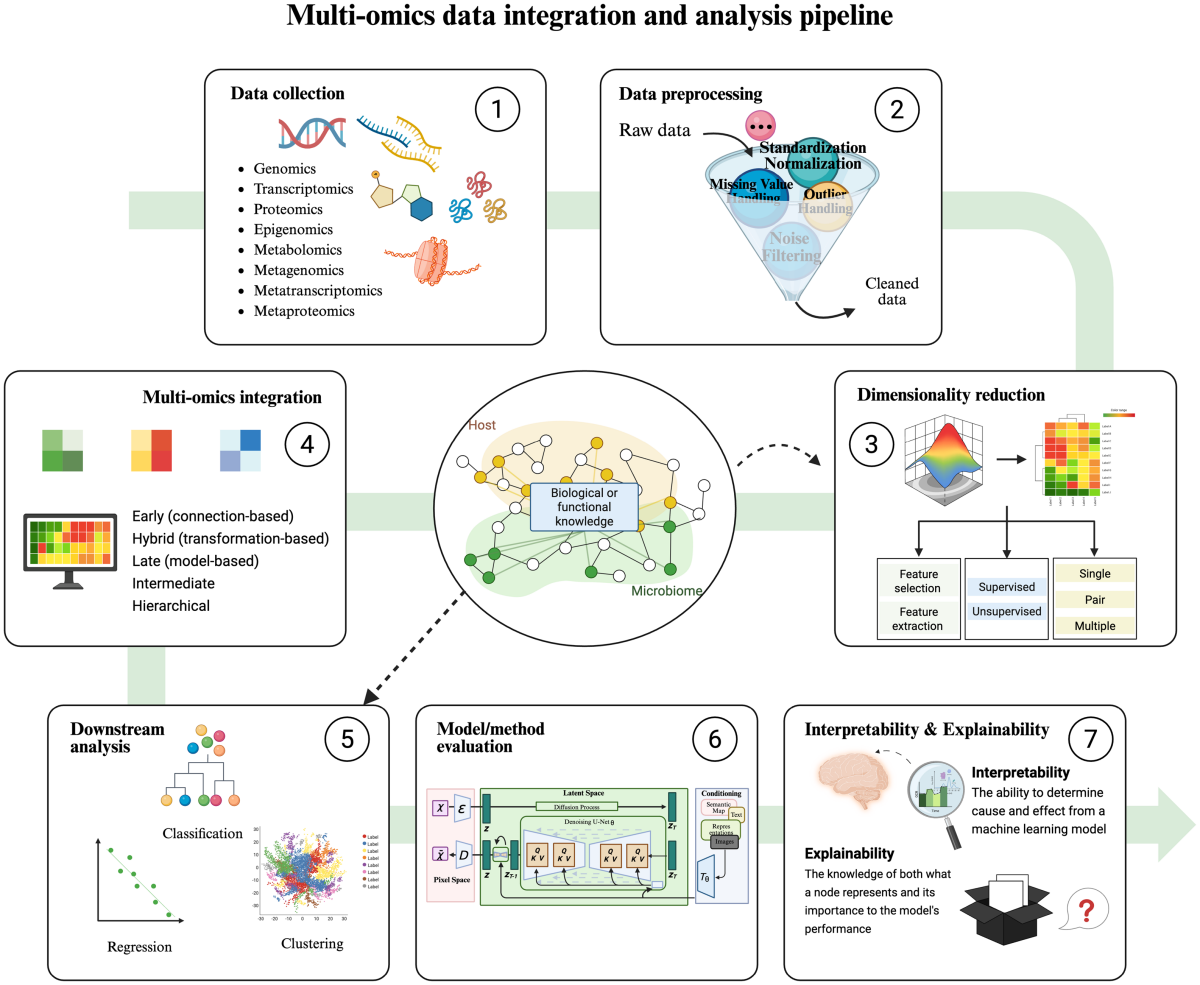
Multi-omics data integration and analysis pipeline. The overall process of integration strategies includes data collection, data preprocessing, dimensionality reduction, multi-omics integration, downstream analysis (classification, regression, clustering), model/method evaluation, interpretability, and explainability. The difference between the Knowledge-Guided Learning Methods and the Data-Driven Methods is that in steps 3 and 5, the former uses a variety of bioinformatics databases related to hosts and microorganisms as a guide to performing data dimensionality reduction, classification, regression, and cluster analysis.

The integration steps of these two methods are primarily similar, as shown in Figure 2, with the key difference in how prior biological information is incorporated into the analysis (Abdelaziz et al., 2024). Data integration strategies are typically divided into horizontal and vertical integration (Zitnik et al., 2019). Horizontal integration involves studying the same omics across different sample groups, while vertical integration examines multiple omics data on the same samples. Vertical integration is more complex and widely applied in practice; the following discussion in this paper focuses on vertical integration.

### 4.3 Dimension reduction of multi-omics integration

Dimensionality reduction is often an essential preprocessing step in multi-omics analysis, as simple integration of omics data may lead to the loss of critical features inherent to each omics layer, exacerbating the “curse of dimensionality” in data integration (Downing and Angelopoulos, 2023). While this step is optional, early and intermediate integration strategies typically require dimensionality reduction to enhance their effectiveness. Standard dimensionality reduction techniques are listed in Table 3.

**Table 3:** Common dimension reduction methods.

Method	Learning approach	Type	Common tools	Advantages	Limitations	Omic datasets
Principal Component Analysis (PCA)	Unsupervised	Feature extraction	base R (R) (Jolliffe and Cadima, 2016); dimRed (R) (Kraemer et al., 2018); mixOmics (R) (Rohart et al., 2017); FactoMineR (R) (Lê et al., 2008); pcaMethods (R) (Stacklies et al., 2007)	Simple, fast, interpretable	Assumes linearity; sensitive to scaling	Single
Independent component analysis (ICA)	Unsupervised	Feature extraction	fastICA (R) (Miettinen et al., 2018); fICA (R) (Miettinen et al., 2018)	Separates mixed signals; extracts non-Gaussian sources	Sensitive to noise; independence assumption may not hold	Single
Multi-dimensional scaling (MDS)	Unsupervised	Feature extraction	base R (R) (Jolliffe and Cadima, 2016); dimRed (R) (Kraemer et al., 2018); MetaboAnalyst	Preserves pairwise distances; effective for visualization	Computationally intensive; sensitive to outliers	Single
Correspondence analysis (CA)	Unsupervised	Feature extraction	vegan (R) (Chen et al., 2024); ade4 (R) (Dray and Dufour, 2024); FactoMineR (R) (Lê et al., 2008); ca (R) (Nenadic and Greenacre, 2007)	Reveals associations in contingency tables	Limited to count/categorical data; interpretation can be subjective	Single
Multiple Factor Analysis (MFA); Hierarchical Multiple Factor Analysis (HMFA)	Unsupervised	Feature extraction	FactoMineR (R) (Lê et al., 2008)	Integrates multiple datasets; balances influence of each data block	Requires careful preprocessing; interpretation may be complex	Single; multiple
Nonnegative Matrix Factorization (NMF)	Unsupervised	Feature extraction	NMF (R) (Wang and Zhang, 2013)	Produces parts-based, interpretable representation	Non-convex optimization; sensitive to initialization	Single
Linear Discriminant Analysis (LDA)	Supervised	Feature extraction	MASS (R) (Xu et al., 2009); caret (R) (Kuhn et al., 2024)	Maximizes class separability; simple and fast	Assumes normality and equal covariance; limited to linear boundaries	Single
Locally linear embedding (LLE)	Unsupervised	Feature extraction	RDRToolbox (R) (Bartenhagen, n.d.)	Captures local structure; reveals non-linear manifolds	Sensitive to noise and parameter settings; computationally heavy	Single
Consensus PCA (cPCA)	Unsupervised	Feature extraction	mogsa (R) (Meng et al., 2016)	Integrates multiple datasets; robust to dataset-specific noise	Less standardized; higher computational demand	Pair
Canonical Correlation Analysis (CCA)	Unsupervised	Feature extraction	CCA (R) (González and Déjean, 2023); vegan (R) (Oksanen et al., 2025); PMA (R) (Chu et al., 2013); mixOmics (R) (Rohart et al., 2017)	Identifies linear relationships between two variable sets	Assumes linearity; sensitive to noise	Pair
Co-inertia analysis (CIA)	Unsupervised	Feature Extraction	made4 (R) (Culhane et al., 2005)	Highlights common structure between datasets	Requires matched samples; interpretation can be complex	Pair
Partial Least Squares (PLS)	Unsupervised	Feature extraction	pls (R) (Mevik and Wehrens, 2007); caret (R) (Kuhn, 2024)	Handles multicollinearity; effective with high-dimensional predictors	The risk of overfitting requires rigorous cross-validation	Pair
Partial Least Squares Discriminant Analysis (PLS-DA)	Supervised	Feature extraction; Feature selection	mixOmics (R) (Rohart et al., 2017); PLS-DA tool (Open source MATLAB tool) (Zontov et al., 2020)	Combines dimensionality reduction with classification	Overfitting risk; model validation is crucial	Pair
Generalized Procrustes Analysis (GPA)	Unsupervised	Feature extraction	Vegan(R) (Oksanen et al., 2025); FactoMineR (R) (Lê et al., 2008)	Aligns multiple datasets; removes scale/rotation differences	Sensitive to outliers; may require iterative convergence	Pair
Multiple co-inertia analysis (mCIA)	Unsupervised	Feature Extraction	omicade4 (R) (Meng et al., 2014)	Simultaneously analyzes several datasets.	Computationally intensive; interpretation is complex	Multiple
Tensor Component Analysis (TCA)	Unsupervised	Feature extraction	tensor BSS (R) (Virta et al., 2024); rTensor (R) (Li et al., 2018); tensorr (R) (Rougier, 2012); ThreeWay (R) (Giordani et al., 2014); SDA4D (R) (Gill and Marchini, 2020)	Captures multi-dimensional interactions; parts-based representation	High computational complexity; challenging parameter tuning	Multiple
Weighted Correlation Network Analysis (WGCNA)	Unsupervised	Feature extraction	WGCNA (R) (Zhang et al., 2021a)	Identifies co-expression modules; robust network construction	Sensitive to parameter selection; high computational cost for large networks	Multiple
Two-way orthogonal PLS (O2PLS)	Supervised	Feature extraction	OmicsPLS (R) (Bouhaddani et al., 2018)	Separates shared vs. dataset-specific variation	Model complexity; challenging interpretation	Multiple
Jint and individual variation explained (JIVE)	Unsupervised	Feature extraction	r.jive (R) (Murden et al., 2022)	Distinguishes shared and unique variation; enhances interpretability	Sensitive to noise; proper model selection is critical	Multiple
Generalized CCA (GCCA)	Unsupervised	Feature extraction	RGCCA (R) (Gloaguen et al., 2022); mixOmics (R) (Rohart et al., 2017)	Integrates more than two datasets simultaneously	High computational demand; similar linearity assumptions as CCA	Multiple
Similarity Network Fusion (SNF)	Supervised	Feature extraction	SNFtool (R) (Burton-Pimentel et al., 2021)	Combines multiple similarity networks; robust to noise	Requires careful tuning of similarity and network parameters	Multiple
Uniform Manifold Approximation and Projection (UMAP)	Unsupervised	Feature extraction	Umap (R) (Yang et al., 2021)	Preserves both local and global structures, faster computation compared to t-SNE	Sensitive to initial conditions, it may not capture all global structures.	Single;Multiple

Dimensionality reduction is typically achieved through feature selection and feature extraction. Feature selection identifies the most representative and informative variables from raw data, such as Recursive Feature Elimination (RFE) (Jeon and Oh, 2020), L1/L2 Regularization (Elastic Net) (Xu et al., 2010), and Least Absolute Shrinkage and Selection Operator (LASSO) (D’Angelo et al., 2009). This approach retains features’ original physical or biological meaning, reduces model complexity, minimizes noise, and lowers the risk of overfitting. However, it may overlook complex feature interactions, potentially missing latent joint information.

In contrast, feature extraction constructs a new feature space, such as through Principal Component Analysis (PCA) (Ringnér, 2008), t-stochastic Neighbor Embedding (t-SNE) (Mrowka and Schmauder, 2024), and Variational Autoencoders (Kingma and Welling, 2019). These methods map high-dimensional data into low-dimensional representations, capturing intrinsic data structures and nonlinear relationships more effectively. Although it achieves efficient information compression, it often results in reduced interpretability.

Depending on whether they utilize data labels, dimensionality reduction techniques can be categorized as supervised or unsupervised. Unsupervised methods, which eliminate redundant variables based on correlations without considering target variables, are suitable for data exploration and structure revelation. Standard unsupervised techniques include Principal Component Analysis (PCA) (Ringnér, 2008), t-stochastic Neighbor Embedding (t-SNE) (Mrowka and Schmauder, 2024), and Nonnegative Matrix Factorization (NMF) (Devarajan, 2008). In contrast, supervised methods leverage known class information to guide the dimensionality reduction process, making them appropriate for classification tasks. Examples of supervised techniques include Linear Discriminant Analysis (LDA) (Ayesha et al., 2020) and Partial Least Squares Discriminant Analysis (PLS-DA) (Lee et al., 2018).

Data integration can be categorized into single-omics, dual-omics, and multi-omics integration, with the number of datasets to be integrated influencing the choice of dimensionality reduction techniques (see Table 3 for details). In practical applications, PCA and LDA can project multi-omics data from the host and microbiome onto a few principal components, retaining the significant variation in the data. This process allows visualization of key gene, protein, and microbiota changes under sepsis conditions (Jonathan et al., 2020). When integrating multi-omics data from different sources, cPCA can eliminate batch effects between datasets and extract standard features reflecting host-microbiome interactions. PLS helps identify host and microbial metabolites that co-vary during disease progression, providing evidence for potential therapeutic targets. WGCNA constructs co-expression or co-abundance networks, clustering related genes or microbes into modules. This step aids in discovering synergistic changes in host genes and microbes at the network level, revealing key regulatory networks and potential biomarkers in sepsis pathogenesis. SNF (Wang et al., 2014) integrates multi-omics data (e.g., genomics, transcriptomics, proteomics) by constructing and fusing similarity networks, capturing shared patterns across omics layers. UMAP reduces the dimensionality of complex host and microbiome multi-omics data for visualization, enabling researchers to intuitively observe data distribution and clustering patterns. Extended CCA integrates multi-omics datasets (e.g., microbiome and metabolome) to capture disease-related multi-omics modules, uncovering significant correlations and highly predictive modules across omics layers (Muller et al., 2024).

### 4.4 Data integration methods and challenges

Due to the problems of high dimensionality, population heterogeneity, and complex data association, it is challenging to utilize the multi-omics data of sepsis effectively. In order to meet these challenges, Iperi et al. (2025) used BiomiX to solve the bottleneck of high-throughput omics data analysis, which can efficiently and integratively analyze multi-omics data from different queues. Moreover, multi-omics factor analysis (MOFA) is commonly used for solving multi-omics data integration (Guo et al., 2025). Besides, direct linkage prioritizes longitudinal multi-omics profiling (e.g., concurrent genomic, metabolomic, and microbiome measurements within identical sepsis cohorts) to reconstruct patient-specific interactomes using advanced integration algorithms (MOFA+/mixOmics). This approach systematically reveals latent covariance structures and time-resolved biological associations. Indirect linkage addresses non-paired datasets through cross-cohort meta-analytic frameworks, statistically harmonizing heterogeneous sepsis cohorts to detect subtle trans-study differential signals.

Three procedures can be employed to address population heterogeneity to mitigate population bias from different data sources. Initially, during the preprocessing phase, the ComBat-harmony algorithm can be applied to eliminate batch effects. Subsequently, a causal inference framework (such as do-calculus) can be introduced in the modeling phase to distinguish confounding factors from accurate biological signals (Dibaeinia et al., 2025). Additionally, transfer learning can bridge the gap between general databases and sepsis-specific data, which is conducive to better handling population heterogeneity across different data sources (Liu et al., 2024). Finally, in the validation phase, when the sample size of specific subgroups is limited, a digital twin cohort can be developed to verify the performance and robustness of the model. Although these methods are reasonable and feasible in theory, adjustments and optimizations are necessary during implementation according to the data and research objectives.

In the context of cross-species (human and animal) data integration and analysis, although the use of phyloP scores effectively identifies conserved molecular features across species (Sullivan et al., 2023), thereby reducing the impact of interspecies differences on research outcomes, we recommend that when collecting data, efforts should be made to ensure similarity between human and animal samples in terms of experimental conditions and processing methods. Data should not be simply combined without consideration; suitable data for aggregation should be carefully selected. Following data cleaning and imputation, the data should undergo standardization and batch effect correction to eliminate systematic differences introduced by different experimental batches or platforms.

Addressing the sparsity issue in omics data, employing deep learning methods such as OmiEmbed (a cross-modal generation approach based on Variational Autoencoders, VAEs) to generate partially missing data represents an advanced strategy (Zhang et al., 2021b). This method can predict missing values based on existing data to address partial missingness in omics datasets. However, when facing comprehensive omics sparsity, leveraging existing biological knowledge to augment data analysis emerges as an effective alternative. Utilizing BioBERT to extract knowledge from literature and constructing virtual omics layers with this knowledge can help mitigate the issue of data sparsity (Lee et al., 2020). Given that deep learning methods and sophisticated statistical models may demand substantial computational resources, the computational cost must be considered when applying these approaches. Moreover, when imputing and integrating data, it is crucial to ensure that the results are biologically meaningful, which can be confirmed through biological validation to verify the reliability of the outcomes.

### 4.5 Types of data integration

Following dimensionality reduction, further analysis and integration of multi-omics data are required. Various integration strategies are available, as proposed by Picard et al. (2021), into five types: early (concatenation-based), mixed (transformation-based), late (model-based), intermediate and hierarchical. Early integration can be selected if each dataset has been preprocessed according to its omics type; this approach involves directly concatenating samples and assembling the resulting matrix as input for machine-learning models. While this method is straightforward, it increases data complexity; therefore, various strategies have been developed to transform or map datasets to facilitate integration. Mixed integration involves independently transforming or mapping each omics dataset, whereas intermediate integration constructs a joint low-dimensional representation across omics. Late integration analyzes each omics dataset separately and aggregates predictions after model training. Hierarchical integration leverages known regulatory relationships between omics, as defined by the central dogma of molecular biology, to integrate datasets.

Numerous tools and platforms based on diverse integration methods have emerged to integrate multi-omics data and investigate host–microbe interactions in sepsis. For example, multi-omics factor analysis (MOFA) and MOFA+ are dimensionality reduction techniques and intermediate integration-based integration tools. These tools take abundance matrices of microbial communities (e.g., bacteria, fungi, viruses) as input and learn low-dimensional representations of samples along with corresponding feature-loading matrices (Argelaguet et al., 2020; Losert et al., 2024). Haak et al. (2021) utilized MOFA to integrate gut microbiota data from sepsis patients and healthy volunteers, including bacterial (16S rRNA), fungal (internal transcribed spacer 1 (ITS1) rRNA), viral (viral metagenomic next-generation sequencing) components and revealed that the proliferation of aerobic pathogens, bacteriophages, and opportunistic yeasts disrupts anaerobic environments, thereby contributing to sepsis pathogenesis.

MintTea (Muller et al., 2024) is an intermediate integration method to identify disease-associated microbial modules from multi-omics data. It elucidates the mechanistic roles of the microbiome in disease and provides evidence for generating system-level, multi-dimensional hypotheses of microbiome-disease interactions. MaAsLin 2 (Mallick et al., 2021) is a tool for multivariable association analysis in microbial community multi-omics studies. It is classified as a late integration method but incorporates features of mixed integration. It is designed to explore associations between microbial community features and complex metadata, such as human health outcomes, diet, and environmental conditions. BZINB-iMMPath (Lin et al., 2023) is a late integration method that constructs metabolite-species and species-species correlation networks. It identifies species modules through similarity-based clustering and facilitates the joint modeling and analysis of microbiome and metabolome data. Zhang S. et al. (2023) introduced a Bayesian modeling method for integrating sparse multivariate count data in microbiome studies. This method leverages joint sparsity to capture feature interactions and supports robust structural estimation in small-sample datasets. Hypergraph-induced orthogonal Nonnegative Matrix Factorization (HONMF) (Ma et al., 2023) is an unsupervised learning framework and an intermediate integration method for microbiome multi-omics data. By integrating three groups of latent variables, it preserves the high-order geometric structure of the original data. It supports sample clustering, data visualization, feature selection, and cross-domain association analysis (e.g., bacteria-virus, fungi-virus interactions). Multimodal Functional Deep Learning for Multi-Omics Data (MFDL) (Zhou et al., 2024) is a deep learning-based intermediate integration method. It integrates various omics data by fitting them into a shared dimensionality-reduction hidden layer at the input level. It enables learning complex relationships between multi-omics data and phenotypes through multi-layer training. EMPress (Cantrell et al., 2021) is an open-source, interactive visualization tool for multi-omics data and employs a late integration approach. It effectively links community-level sample groupings with feature-level structures, supporting exploratory analysis of complex multi-omics datasets.

### 4.6 Guidelines for clinical sample collection in sepsis

One crucial aspect is that researchers need to minimize sample heterogeneity when collecting samples. Factors to be considered include chemotherapy, active or passive immunotherapy, antibiotic medication use, lifestyle, etc., which can disrupt the microbial balance and consequently damage the network within the bacterial community and its relationship with the host.

The sepsis-specific biobank was designed, and different sampling time windows, core omics layer, and extended omics layer were recommended for different stages of sepsis. During the early recognition phase (0–6 h), it is suggested to collect metabolomics (plasma) and microbiome (fecal) samples within the first hour of initial presentation in the emergency department, along with single-cell transcriptomics (peripheral blood mononuclear cells, PBMC) analysis. Metabolomics and microbiome samples are relatively inexpensive and easy to collect, with high clinical feasibility and cost-effectiveness. During the progression phase (6–72 h), dynamic sampling of proteomics (serum) and lipidomics (plasma) every 12 h is recommended. Although these samples are easy to collect, they require substantial resources and incur higher costs. Spatial transcriptomics (tissue biopsy) is more challenging to sample but offers deeper analytical insights. In the recovery or sequelae phase, follow-up visits are recommended at 30, 90, and 180 days post-discharge to collect circulating cell-free DNA (cfDNA) and exosome omics samples and perform longitudinal analysis of gut metagenomics. This stratified sampling strategy facilitates comprehensive acquisition of the multi-omics features of sepsis, providing critical evidence for early disease recognition, progression monitoring, and prognostic assessment.

In order to reduce costs, some intensive care centers are equipped with core devices such as mass spectrometers and sequencers to meet the real-time decision-making needs for septic shock. An automated diagnostic platform is constructed and can be operated by grassroots medical staff. At the same time, data analysis is automatically completed by cloud-based learning models, thereby achieving intensive and efficient use of resources.

## 5 Application of integrative omics in the diagnosis and treatment of sepsis

### 5.1 Discovery of diagnostic markers

Since infection and host-pathogen interactions constitute a complex nonlinear system, no single biomarker can accurately diagnose sepsis (Pierrakos et al., 2020). Multi-omics integration offers new avenues for identifying diagnostic markers of sepsis. Metagenomic sequencing and 16S rRNA analyses reveal altered gut microbiota structure and function in sepsis patients. Specific microbes (e.g., *Enterococcus* abundance) and metabolites (e.g., SCFA levels) are linked to sepsis progression and hold potential as diagnostic markers. Integrating host omics data, such as inflammatory gene expression in the host transcriptome and specific protein markers in the proteome, with microbiome data enhances diagnostic accuracy and specificity (Malik et al., 2024).

In a multicenter prospective study (van Houten et al., 2018), researchers collected blood, nasal swabs, and fecal samples from sepsis and non-infected individuals. Multi-omics analyses included host RNA and protein biomarker detection, nasal and gut microbiota analysis, host genomics, and bacterial proteomics. A multi-parameter model was developed to distinguish between bacterial and viral infection etiologies. This study provided a diagnostic tool for differentiating bacterial and viral infections and also offered data support for bacterial subtype classification, providing a more accurate basis for sepsis diagnosis. Ma et al. (2022) integrated host transcriptomics, microbiomics, and metabolomics data and found that in cecal sepsis caused by methicillin-resistant *Staphylococcus aureus,* CYP1A1 deficiency improved gut barrier function and reduced the accumulation of the harmful metabolite cadaverine, whose level was positively correlated with the clinical SOFA score, and could offer new biomarkers for early sepsis diagnosis and treatment. Using the BZINB model, an integrative multi-omics model, disease-related modules in the microbiome and metabolome were identified. Specific correlations between certain metabolites and microbes were found in healthy and orally diseased patients, providing new insights for developing disease diagnostic biomarkers (Lin et al., 2023).

### 5.2 Development of personalized treatment strategies

Integrating multi-omics technologies enables in-depth analysis of patients’ genomics, transcriptomics, proteomics, metabolomics, and gut microbiomes. This approach precisely characterizes each patient’s unique pathophysiological profiles, immune status, microbial composition, and interactions, thereby enabling the gradual realization of personalized medicine in sepsis therapy.

If a deficiency of beneficial gut microbiota or an overgrowth of pathogenic bacteria is detected, interventions such as probiotic supplementation, prebiotic administration, or fecal microbiota transplantation (FMT) can be employed (Lisko et al., 2017). For instance, FMT has been shown to restore and increase butyrate levels via the IRF3/NF-κB signaling pathway, counteract the immunosuppressive effects of sepsis pathogens, and improve sepsis-induced muscle atrophy (Kim et al., 2020). By integrating host genomic and transcriptomic data, it is possible to predict patient responses to medications, select more appropriate antimicrobial agents, prevent antibiotic misuse, and achieve precision medicine. Additionally, dynamic monitoring based on omics data can facilitate timely adjustments in therapeutic strategies, thereby enhancing treatment outcomes and improving patient prognosis. Yang et al. (2024) utilized a two-sample bidirectional Mendelian randomization analysis, integrating data from genome-wide association studies (GWAS), eQTL datasets, single-cell transcriptomics, and large-scale RNA sequencing. By combining insights into gut microbiota regulatory mechanisms with drug databases, they precisely evaluated the association between gut microbiota and sepsis, identified potential genes and targets, and deeply explored and validated potential therapeutic agents for sepsis. Mu et al. (2023) used clinical isolates of four sepsis-causing bacterial strains and integrated multi-omics data, including genomics, transcriptomics, proteomics, and metabolomics, to investigate the responses of sepsis pathogens in a human serum environment. Their findings provide critical evidence for the development of therapeutic targets for sepsis. Li D. et al. (2024) conducted bulk RNA sequencing, single-cell transcriptomic analysis, metabolomics, and 16S rDNA sequencing of gut microbiota using peritoneal lavage fluid (PLF) cells in mice. They discovered that rhamnose derived from the gut or therapies targeting SLC12A4 may enhance macrophage phagocytosis during sepsis, offering a novel potential direction for clinical sepsis treatment.

### 5.3 Therapeutic effect monitoring and prognosis evaluation

In the treatment of sepsis, multi-omics technologies play a critical role. By integrating and analyzing multi-omics data, including the microbiome, host transcriptome, proteome, and metabolome, these technologies enable real-time and comprehensive monitoring of therapeutic efficacy, provide deep insights into disease progression, predict patient outcomes, and offer a scientific basis for formulating and adjusting treatment plans with precision. This approach ultimately contributes to improving clinical outcomes for patients with sepsis.

In treating neonatal sepsis, monitoring the expression changes of *Staphylococcus epidermidis* virulence genes and the dynamics of transcriptional regulatory networks, combined with the unique host environmental factors of neonates, comprehensively evaluates the role of treatment in controlling infection and improving prognosis. Providing key information for optimizing subsequent treatment decisions and, more precisely, grasping the progress and direction of sepsis treatment (Joubert et al., 2022). Using the central security database (HoPOIT database) to standardize, integrate, and dynamically monitor multi-omics data of patients (such as host biomarkers, pathogen characteristics, and drug resistance information), the temporal dynamics of host-pathogen interactions are analyzed, revealing the changing trends of biomarkers during treatment (van Houten et al., 2018). Sepsis patients are categorized into different enterotypes by integrating gut microbiota 16S rRNA analysis and metabolomics. It is found that patients with enterotype E3 have the most severe conditions. The OTU773 of *Bacteroidota* and OTU822 from *Rikenellaceae* are significantly positively correlated with ICU length of stay. 5-Hydroxyindoleacetylglycine is positively correlated with the APACHE II score, and three compounds negatively correlate with ICU length of stay. Long et al. (2023) utilized metagenomics and metabolomics to delineate the dynamic changes in gut microbiota and their metabolites in sepsis patients at different stages of ICU admission. They observed that in sepsis patients, gut microbiota diversity, the relative abundance of *Firmicutes*, and SCFA levels were significantly reduced, whereas the relative abundance of *Proteobacteria* and primary bile acid levels markedly increased with prolonged hospitalization. Among the differential microbiota and metabolites, the relative abundance of *Klebsiella* and the concentrations of butyrate and taurocholic acid exhibited strong correlations with sepsis patient prognosis, providing direction for prognostic evaluation and the adjustment of treatment plans based on microbiota profiles. These findings indicate that alterations in gut microbiota and metabolites are associated with sepsis’s progression and clinical outcomes, providing a basis for early prediction of clinical outcomes and exploration of new therapies (Sun et al., 2023).

## 6 Current challenges and future directions

In the field of sepsis, multi-omics integration has achieved some progress in exploring host-microbiota interactions, but many challenges remain, and there is still considerable room for development. From the perspective of omics technology development, novel technologies such as single-cell and spatial omics are continuously emerging and evolving. Single-cell RNA sequencing (scRNA-seq) (Cheng et al., 2023) and single-cell nascent RNA sequencing (scGRO–seq) (Mahat et al., 2024) can reveal cellular heterogeneity at the single-cell level and provide deeper insights into cellular functions and characteristics. Spatial omics, on the other hand, allow for the investigation of cellular microenvironments and intercellular interactions while preserving the spatial structure of tissues (Wang J. et al., 2024). Currently, Janosevic et al. (2021) used scRNA-seq to detect changes in various renal cell populations in septic mice during the disease process and combined spatial omics sequencing to provide a spatiotemporal dynamic map of septic kidneys at the cellular and molecular levels. However, there are currently no studies on the relationship between microbiota and host in sepsis at the single-cell and spatial levels. Integrating emerging omics technologies to locate the distribution of microbiota within the host precisely and their impact on surrounding tissues and cells, as well as deeply exploring the spatial interactions between host and microbiota in sepsis, holds great potential.

From the perspective of the disease process, most existing multi-omics studies are focused on static analysis, that is, testing samples at specific time points. However, sepsis is a dynamically evolving pathological process, with microbial community structures, host gene expression, and metabolite levels all fluctuating continuously over time. The lack of dynamic monitoring limits our understanding of critical turning points and intervention timing during the disease process, making it difficult to provide precise time-window guidance for precision treatment. Dynamic modeling approaches should be adopted to study bacterial populations from a dynamic perspective and integrate data from multiple time points or conditions.
